# Association of myeloid cell reactivity patterns with safe food predictions in FPIES patients

**DOI:** 10.1186/s13223-025-00968-1

**Published:** 2025-05-21

**Authors:** Georgiana M. Sanders, Alexandra Hua, Elizabeth Hudson, Jonathan P. Troost, Nobuhiko Kamada, John Y. Kao, Charles F. Schuler, Mohamad El-Zaatari

**Affiliations:** 1 Division of Allergy and Immunology, Department of Internal Medicine, University of Michigan, 24 Frank Lloyd Wright Drive, Ann Arbor, MI 48105 USA; 2 Mary H. Weiser Food Allergy Center, University of Michigan, 24 Frank Lloyd Wright Drive, Ann Arbor, MI 48105 USA; 3 Department of Internal Medicine, University of Michigan, Ann Arbor, MI USA; 4 Michigan Institute for Clinical and Health Research, University of Michigan, Ann Arbor, MI USA; 5 Division of Gastroenterology and Hepatology, Department of Internal Medicine, University of Michigan, 6518 MSRB 1, 1150 W. Medical Center Drive, Ann Arbor, MI 48109 USA

**Keywords:** Non-IgE food allergy, FPIES, Food allergy, Gastrointestinal inflammation, Vomiting, Diarrhea

## Abstract

**Background:**

Food protein-induced enterocolitis syndrome (FPIES) is an understudied non-IgE-mediated food allergy, which is distinct from and lacks diagnostic testing akin to IgE testing. FPIES affects infants and toddlers but can persist into adulthood. As there are no extant methods to identify safe foods for FPIES patients, food ingestion trials are performed at home and often lead to reactions and development of food aversions, which may lead to failure-to-thrive and gastric feeding tube requirements. We hypothesized that foods that fail to elicit responses in immune cells of FPIES patients would be safe to ingest, which could support development of a diagnostic method to headstart safe food identification in patients.

**Methods:**

We developed an ex vivo model of FPIES using food-stimulated white blood cells (WBCs) from pediatric FPIES patients and controls by defining a 9-gene panel representative of FPIES ex vivo responses and conducted a single-arm pilot clinical trial.

**Results:**

Myeloid cells of FPIES patients displayed variable individual-specific myeloid cell reactivity patterns (iMCRPs) to different foods. Foods that failed to elicit repsonses in patients’ immune cells were safe to ingest with a negative predictive value of 98.5%. This, when utilized in prospective predictions, reduced newly introduced food reaction rates from 19.5 to 0% while increasing food repertoire diversity.

**Conclusions:**

iMCRPs represent a novel and potentially useful tool that associates with safe food ingestion in FPIES patients for foods that fail to elicit immune cell reactions.

*Trial Registration* The trial has been registered at registered at ClinicalTrials.gov # NCT04644783.

**Supplementary Information:**

The online version contains supplementary material available at 10.1186/s13223-025-00968-1.

## Background

Non-IgE-mediated food allergies are a diverse family of immunologic adverse food reactions that do not involve IgE [1]. Non-IgE food allergies include food protein-induced enterocolitis syndrome (FPIES), food protein-induced allergic proctocolitis, and eosinophilic esophagitis [2]. Their mechanisms remain incompletely understood [2]. As a result, no definitive diagnostic tests or biomarkers for non-IgE food allergies exist. Typical IgE-type allergy testing, which relies on detecting IgE antibodies via food skin or blood-based testing methods, cannot distinguish foods that are safe to eat versus those that trigger reactions in these conditions [1].

FPIES is a relatively prevalent type of non-IgE food allergy [1] characterized by severe repetitive vomiting reactions to specific foods 1–4 h after ingestion [1, 3]. FPIES affects over 900,000 people in the United States [4], primarily infants and toddlers under age three [1, 5]. Foods that cause reactions are called “trigger” foods, while foods that do not are called “safe” foods [6]. Although population studies suggest that some foods pose a higher risk to trigger an FPIES reaction, any food can be a trigger [6], and work from our own group suggests that the risk profile of FPIES triggers in the population might change over time [7].

Foods never previously ingested must be “trialed” because there is no existing method to predict if a new food will be safe [1]. Food ingestion trials are performed at home without any testing using “low-risk” foods based on FPIES population data [1]. Unfortunately, numerous reactions occur during these essentially random food trials [7, 8]. This traumatizes children and families and often leads to profound anxiety, food aversions, gastric feeding tube requirements, developmental delays, severe growth and nutritional deficiencies, and/or reduced quality of life [9–14]. Moreover, the manner in which foods are introduced following standard-of-care (SOC) recommendations is restrictive because patients are advised to trial only low-risk foods initially and to delay high-risk food introductions [1]. This approach may reduce patients’ food repertoires at a critical phase in human development [15].

Initial stages of FPIES after diagnosis, when few or no safe foods had been identified, particularly pose a challenge to patients when the diet is still highly restricted. Therefore, there is an urgent need to develop novel methodology to identify a number of individual-specific safe foods during the early stages of the disease, to provide a sufficient repertoire of foods and circumvent food aversions leading to failure-to-thrive from ensuing.

Development of novel diagnostics is limited by a need for a better understanding of FPIES pathophysiology. While serotonin signaling [16], innate immunity [17], and IL-17-related signalling [18] are implicated in the disease pathophysiology based on real-life FPIES reaction sampling, no model systems exist in which to study the disease. In the present study, we developed an ex vivo model of FPIES reactions using peripheral WBCs to define an RNA-based panel of FPIES response genes. We identified that the major source of direct FPIES immune cell responses to food stimulation occurs within myeloid cells. We utilized this ex vivo model as a diagnostic approach to identify a number of safe foods in FPIES and conducted a pilot clinical trial to provide preliminary validation of this model’s performance.

## Methods

### Study approval

The study was approved by the Institutional Review Board (IRB) at the University of Michigan under IRB protocol # HUM00156027. Written informed consent was signed for by one one of the participants’ parents or guardians. Recruitment of FPIES participants was conducted under a single-arm clinical trial of 10 FPIES participants, registered at ClinicalTrials.gov (NCT04644783). Blood samples from 5 de-identified non-FPIES participants were collected independently of the clinical trial, serving as an external control arm under IRB protocol #HUM00208717.

### Participant recruitment

Participants from the FPIES clinical trial were contacted by email and/or telephone after pre-screening physician-diagnosed FPIES patients who had recently visited the UM allergy clinics. Inclusion criteria comprised patients aged 1 month to 3 years old with a physician diagnosis of FPIES, and with documented reactions to 2 or more trigger foods with recurrent delayed vomiting. Exclusion criteria included: use of medications that suppress the immune system, individuals without at least 2 FPIES trigger foods identified, any history of another GI disease (e.g., inflammatory bowel disease, celiac disease, etc.), cardiac, pulmonary, neurologic, renal, endocrine, or gynecological pathology, and/or lack of parental/guardian informed consent.

The parents/guardians were asked to complete Questionnaire 1, which listed their known safe and trigger foods, at the time of entry into the study. A blood draw was then scheduled, and the patients were instructed to only consume known safe foods for one week prior to the blood draw.

### Diet management between blood draw and completion of assay results

Between the blood draw and assay completion, the allergist and nutritionist managed participants’ food introductions. The participants were instructed to complete Questionnaire 2 with detailed information about all the foods introduced until the results of the assay were received.

### Standard of care comparison group

Secondary data for a “standard of care” (SOC) group were collected from a prior study collected by our group [7]. The original dataset comprised 347 children with a diagnosis of FPIES seen in the University of Michigan Allergy and Immunology clinics from 2010–2012. Full details on the cohort are available in our prior publication [7]. For the present work, we selected a comparison group consisting of individuals with two or more FPIES trigger foods who had an FPIES age-of-onset between 4 and 10 months to correspond to the participants enrolled in the prospective trial above.

### Assay predictions and recording of outcomes

When assay results were available, the assay predictions for safe foods were relayed to the participants’ parents/guardians, who were instructed to trial the predicted safe foods. The participants’ parents/guardians completed Questionnaire 3, which reported the outcomes of each food trial from the safe food prediction list based on assay predictions.

### Blood collection and white blood cell (WBC) culture

To perform the assay, 6 ml blood samples were collected in K2 EDTA (lavender top) blood tube from each FPIES participant at clinical phlebotomy stations at Michigan Medicine by specialized phlebotomists. For non-FPIES controls, the blood was obtained from lead testing samples from de-identified participants. The blood was treated with ACK buffer to lyse the red blood cells, and the total WBCs were plated in a 12-well plate and cultured in RPMI + 10% FBS at 37 °C and 5% CO2, and then treated/saturated for 3 h with ~ 1 mg/ml freshly prepared food homogenate, 2 μg/ml LPS, or left untreated. After 3 h, the media was removed and the cells lysed in buffer RLT (Qiagen, Germantown, MD) with β-mercaptoethanol, homogenized using QIAshredder (Qiagen), and the RNA extracted using the RNEasy Microkit (Qiagen). RT-qPCR was performed using SuperScript III Reverse Transcriptase (Invitrogen/Thermo Fisher Scientific, Massachussets) and Platinum Taq DNA polymerase (Invitrogen/Thermo Fisher Scientific).

### Prospective safe food predictions

The fold change expression values for each gene of the 9-gene panel, relative to the untreated, were cumulatively added. These values were calculated in response to each food treatment and then divided by the cumulative value obtained for the 9-gene panel in response to LPS, and then multiplied by 1,000. These values were plotted for foods that had been retrospectively identified as triggers, which were labeled as “retrospective triggers”, and foods that had been retrospectively identified as safe foods, which were labeled “retrospective safe’s”. These expression values identified a cut-off value of 29.24, at which the NPV was 98.5, which corresponded to a 98.5% chance for a food, of unknown status, to be safe when its expression value of the 9-gene panel normalized to LPS was below 29.24. Foods of unknown safe or trigger status with an expression value below 29.24 were therefore prospectively predicted to be safe, and the list of such foods was communicated to the FPIES participant parent/guardian.

### Microarrays and microarray analyses

Microarrays were performed by the University of Michigan Advanced Genomics Core, and the analyses were performed by the Bioinformatics Core at the University of Michigan. Genes were ordered by the highest to lowest induced genes in response to trigger foods relative to safe foods.

### Clustering and plotting in 2 dimensions

K-means, which is a clustering algorithm that partitions the samples into k groups by minimizing the sum of squares between each sample in a cluster and its center, was run using k = 2 on the panels of 208, 30, and 9 genes. To plot the k-means results in 2 dimensions, the “fviz_cluster” method from the factoextra R package (version 1.0.7) was used. The method takes the expression data and the k-means clustering result and plots the first two principal components with the clusters colored.

### Single-cell RNA sequencing (scRNA-Seq) analyses

Single-cell RNA sequencing and processing (read alignment and counting) using cell ranger were performed by the Advanced Genomics Core, and scRNA-Seq analyses by the Bioinformatics Core at the University of Michigan. Clustering and sub-clustering were performed following the Seurat integration and clustering workflow: https://satijalab.org/seurat/articles/sctransform_v2_vignette.html. The count data from the 9 samples [2 untreated (1 from each participant), 2 LPS-treated (1 from each participant), 2 safe food-treated (1 from each participant), and 3 trigger food-treated (1 trigger food treatment from one participant, and 2 trigger food treatments from the other participant)] were normalized using SCTransform and integrated into one object using SelectIntegrationFeatures (which selected 3000 variable genes used for integration and PCA), PrepSCTIntegration, FindIntegrationAnchors, and IntegrateData. Then clustering was performed using RunPCA (using the variable genes), FindNeighbors (based on the first 30 dimensions learned in PCA), and FindClusters (resolution = 0.2). UMAP embeddings were generated using RunUMAP.

### SYK, TLR4, TLR2 and MYD88 inhibition

Total WBCs were cultured from one participant and treated with rice, which was a trigger food for that individual that elicited a response within the 9-gene panel, relative to untreated. Rice treatments were administered to the WBCs in separate wells of a 12-well plate after the following 2-h pre-incubations with: (i) SYK inhibitor (R406, cat# inhr406; Invivogen, San Diego), (ii) TLR4 inhibitor (VIPER, cat# NBP2-26,244; Novus Biologicals, Colorado), (iii) TLR2/4 inhibitor (TIRAP inhibitor; cat# NBP2-26,245; Novus Biologicals), and (iv) MyD88 inhibitor (homodimerization Inhibitor peptide; cat# NBP2-29,328; Novus Biologicals). Inhibitor control peptides were utilized for each inhibitor as supplied, by the manufacturer, with each of the inhibitors.

### Biostatistical analyses

Biostatistical analyses were performed by the Applied Biostatistical Sciences (ABS) Network at the Michigan Institute for Clinical and Health Research (MICHR). The primary outcome measure for the clinical study was NPV, defined as the proportion of test-predicted safe foods that were physiologically confirmed as safe foods. A random-effects logit model was used to model the binary outcome (safe or trigger food) as a function of the 9 biomarker measurements in the assay. The random effect in the logit model took into consideration the correlated data measured within the same participant. A cluster Receiver operating characteristic (ROC) curve analysis was used to assess the precision of the assay. Specifically, the area under the cluster ROC curve (AUC), along with a 95% confidence interval (CI), was calculated. The assay was considered predictive if the lower limit of the 95% CI is above 0.5, which is the null value indicating no predictive ability.

### Data availability

The sc-RNA-Seq data presented in this study has been deposited in NCBI's Gene Expression Omnibus (GEO): https://www.ncbi.nlm.nih.gov/geo/query/acc.cgi?acc=GSE232839.

### Sex as a biological variable

The study accounted for sex as a biological variable by including data from 6 females and 4 males.

## Results

### Identification of 9-gene panel associated with FPIES trigger food treatment in a case study.

**Fig. 1:**
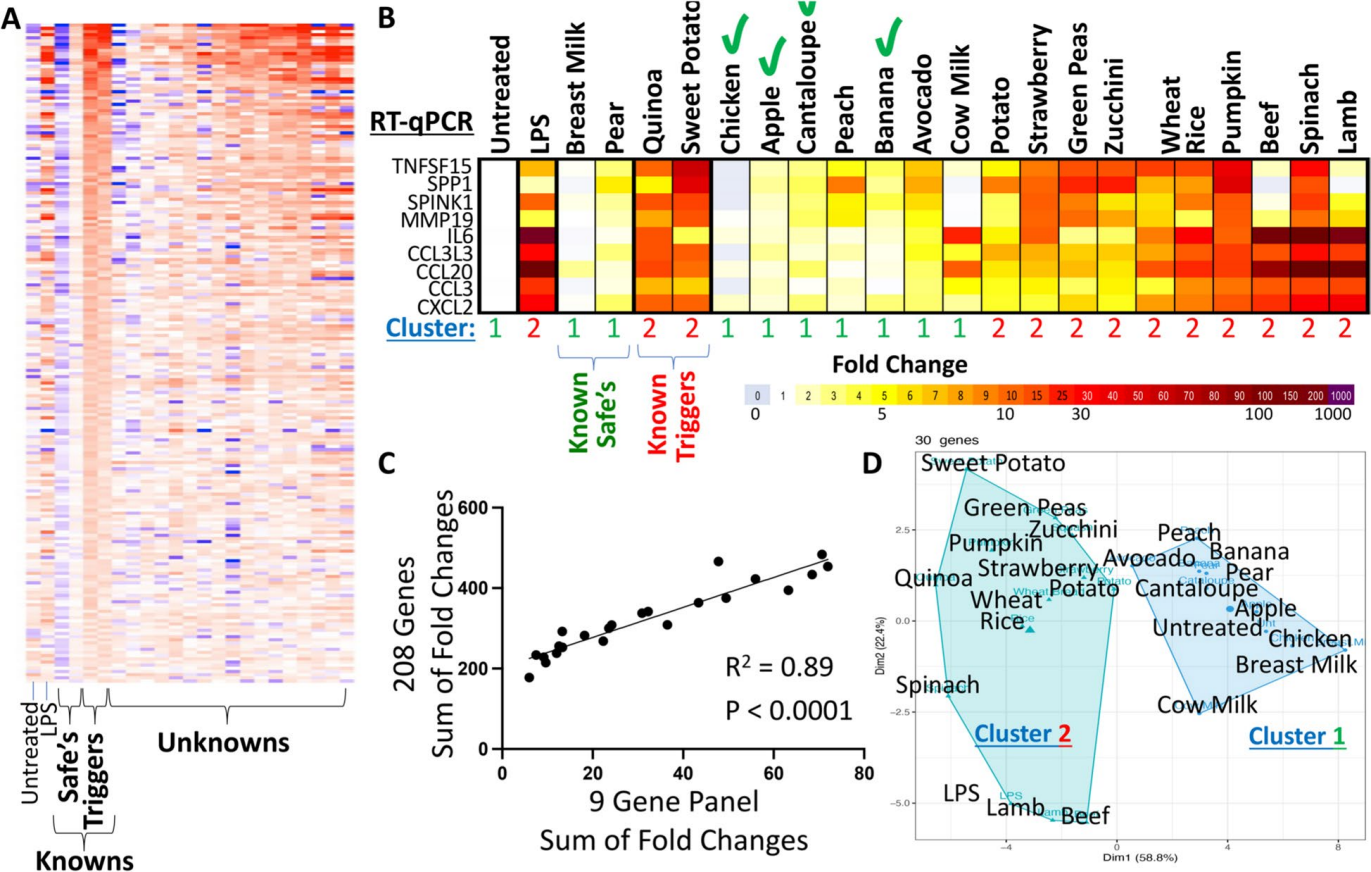
Case study identifies 9-gene panel associated with FPIES triggers. **A** Microarray heatmap of 208 genes that are induced by treating FPIES patient’s WBCs ex vivo with 2 known triggers versus 2 known safe foods. Each column represents one treatment including untreated, LPS, 2 known safe foods, 2 known trigger foods, and individual food treatments of unknown safe or trigger status. **B** 9-gene RT-qPCR panel representative of the gene induction in (**A**). **C** Correlation of the sum of expression of the 9-gene panel vs the 208-gene panel. Each data point represents one treatment comprising untreated, LPS, or individual food treatments. **D** Clustering of foods by a k-means clustering algorithm, for the 30 highest induced genes by the two trigger foods, separates a response-lacking food cluster (Cluster 1) from a response-triggering food cluster (Cluster 2). The two clusters are also listed under the heatmap in (**B**)

Given the innate immune contribution to FPIES reactions previously reported [17], we sought – via a case study – to identify the nature of the innate immune cells that respond to food treatments that trigger a reaction. We utilized total white blood cells (WBCs), from peripheral blood from the case study individual, since these encompass various types of immune cells, and treated them ex vivo with two known trigger foods, which had previously elicited a reaction in the case study subject, versus two known safe foods, which had not elicited a reaction. We also evaluated 17 individual food treatments with no history of ingestion and thus of unknown FPIES reaction status. Untreated and lipopolysaccharide (LPS)-treated WBCs were used as controls. Using transcriptomics, we identified a panel of 208 genes that were induced by the two known trigger foods but not by the two known safe foods (Fig. 1A and Supplementary Fig. 1). We simplified the gene list to a 9-gene panel that was robustly detectable by RT-qPCR (Fig. 1B), and which strongly correlated with the expression of the 208 gene panel (Fig. 1C; each datapoint represents one treatment), indicating that this abridged panel was representative of the global transcriptomic changes per food treatment. Clustering of the responses to different food treatments by a k-means clustering algorithm for the highest 30-induced genes distinctly separated food classifications into 2 clusters: [1] a response-lacking food cluster (Cluster 1; Fig. 1B, D), versus [2] a response-triggering food cluster (Cluster 2; Fig. 1B, D), indicating that, irrespective of the nature of the food treatment, the cellular transcriptional responses were similar when induced.

To test the hypothesis that response-lacking foods were safe to ingest, the case study participant prospectively trialed the new foods with RNA profiles akin to known safe foods. These new foods were found to be safe (Fig. 1B; green check marks). Two additional panels for safe food predictions were repeated for the case study individual without the occurrence of further FPIES reactions (data not shown).

### Corroboration of the association of the 9-gene panel with FPIES reactions.

**Fig. 2:**
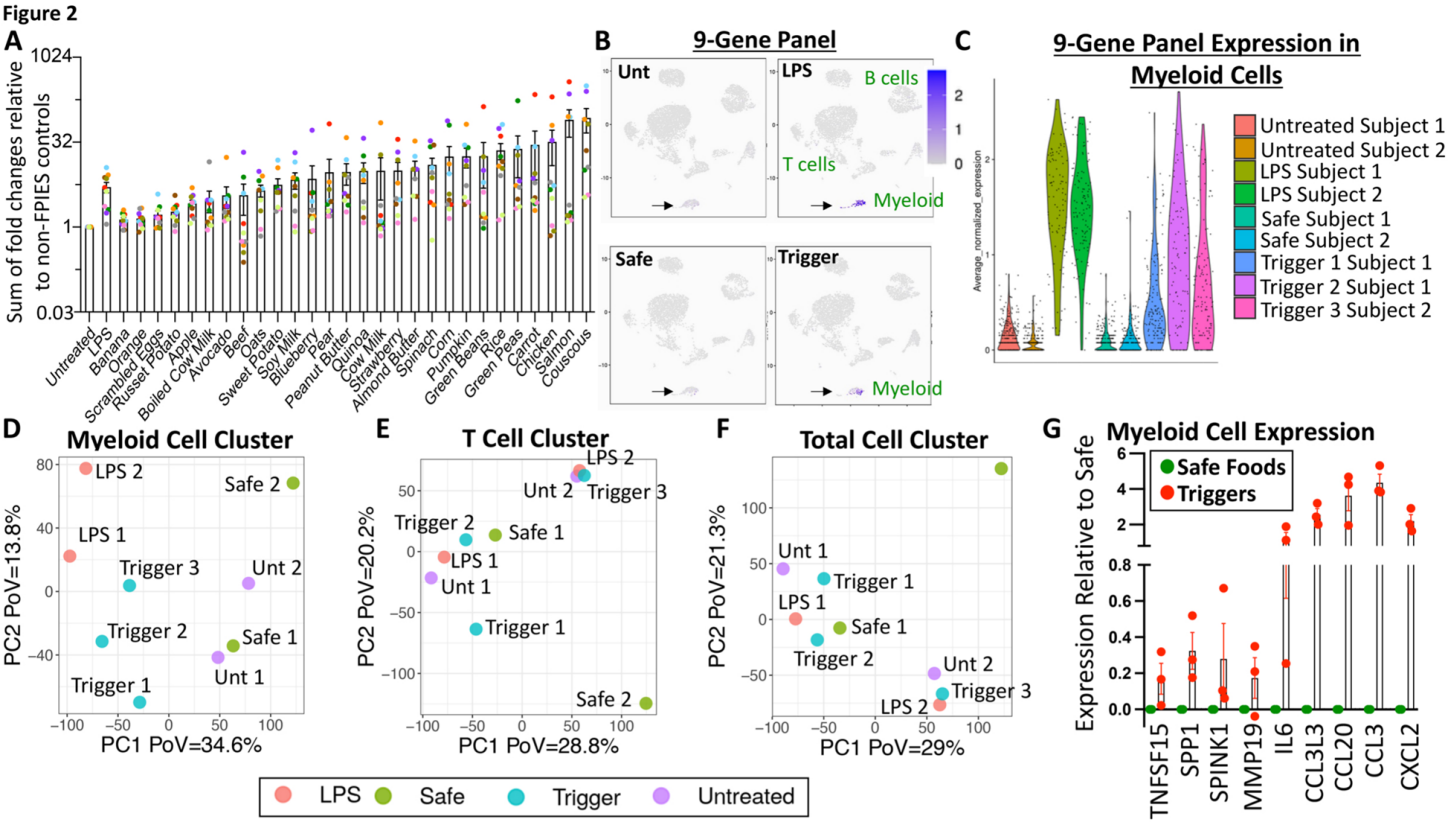
*Corroboration of the association of the 9-gene panel with FPIES* reactions. **A** Different FPIES participants exhibit individual-specific inter-participant variability in their WBC response to the same food treatments. Each data point per treatment represents one participant. Values are normalized to the responses in control non-FPIES participants. **B** UMAP clustering showing induction of the 9-gene panel in myeloid cells in response to LPS or trigger food stimulation (black arrow), by scRNA-Seq. **C** Violin plots of the 9-gene panel (scRNA-Seq) expression in myeloid cells. **D**-**F** PCA plots showing the clustering of genes following untreated, safe food, trigger food, or LPS treatment after clustering for myeloid cell genes (**D**), T cell genes (**E**), or total cell genes (**F**). **G** Corroboration of the induction of each gene of the 9-gene panel by scRNA-Seq from 2 safe food treatments versus 3 trigger food treatments from 2 patients

To assess the significance and corroborate the relevance of the 9-gene RT-qPCR panel that we had identified, we conducted a single-arm clinical trial of 10 FPIES participants (Supplementary Fig. 2) with an external control arm of 5 non-FPIES controls (with equivalent ages to the study participants, from de-identified lead screening samples) (Supplementary Fig. 2A). We compared the expression of the 9-gene panel between different FPIES participants, normalized to non-FPIES controls in response to different food treatments, using a fixed panel of 28 foods for all ex vivo WBC treatments (Fig. 2A; each data point, with a distinct color, represents one participant; responses are normalized to the responses of non-FPIES controls per food). We evaluated the variability in WBC responses to the same food treatments between participants, and observed that these responses varied in an individual-specific manner (Fig. 2A). We confirmed that this variability was not due to intra-assay variability within individuals by repeating the test from different blood draws from the same individual on two different days and obtaining similar responses (Supplementary Fig. 3).

Two participants were selected to evaluate the induction of the 9-gene panel by FPIES triggers in greater detail via scRNA-Seq. We selected 3 response-triggering foods from the 2 participants, which were known trigger foods for those individuals, and 2 response-lacking foods that were known safe foods. We observed by scRNA-Seq that the induction of the genes from the 9-gene panel was specific to myeloid cells (Fig. 2B, C). Principal component analysis (PCA) plots showed that the clustering by gene expression for the myeloid cell sub-cluster segregated the untreated and safe food groups from the LPS and trigger food groups (Fig. 2D), whereas the T cell sub-cluster or total cell cluster failed to segregate the foods as such (Fig. 2E, F). Finally, scRNA-Seq from the pooled data in these 2 participants corroborated the induction of each gene of the 9-gene panel within the myeloid cell sub-cluster (Fig. 2G). These data indicated that the responding cells to short-term food stimulation were myeloid cells, and not T cells. We termed the individual-specific myeloid cell response patterns to different foods “individual-specific myeloid cell reactivity patterns” (iMCRPs). We conclude, based on the microarray and scRNA-Seq data, that iMCRPs of FPIES participants correlate with trigger food induction.

### Predictive capacity of 9-gene panel for safe foods in FPIES participants.

**Fig. 3:**
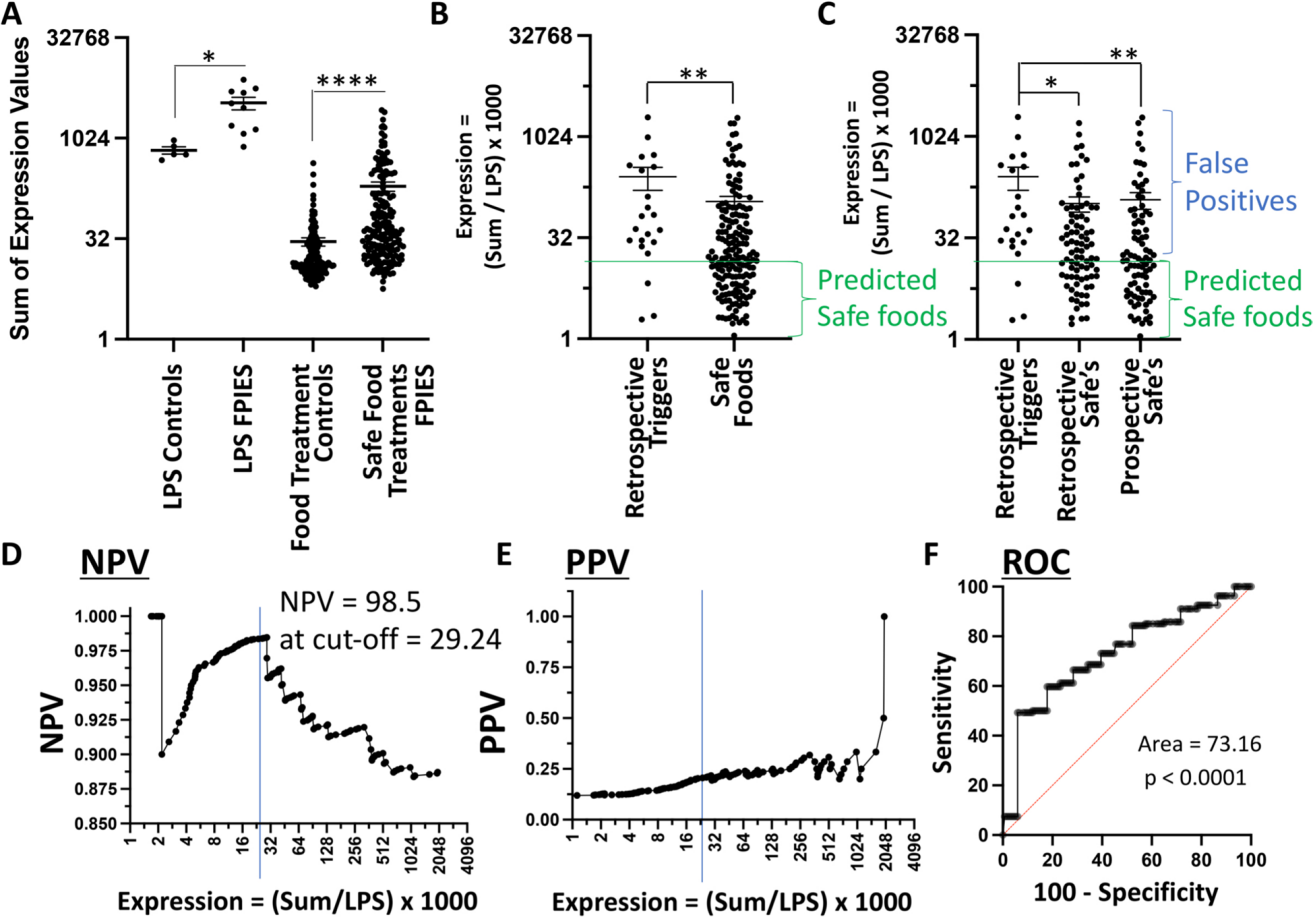
Prediction of safe foods for FPIES participants. **A** Expression values of the 9-gene panel of FPIES participants’ WBC responses to LPS or safe food treatments relative to non-FPIES control participants’ WBCs. The data points are representative of pooled responses from 10 FPIES participants relative to 5 non-FPIES controls **B**, **C** Expression values from the 9-gene panel in safe versus trigger foods pooled from 10 FPIES participants and normalized to LPS. The NPV threshold of 29.24 for safe food predictions is annotated. Foods known to be safe retrospectively versus prospective safe food predictions are stratified in (**C**). ****p < 0.0001; **p < 0.01; *p < 0.05. **D** Graph representing NPV values relative to expression values. The optimal cutoff expression value of 29.3 for an NPV value of 98.5% is annotated. **E** Graph representing PPV values. **F** ROC curve. ****p < 0.0001

To assess the predictive capacity of the assay, we first established the baseline responsiveness in FPIES participants versus controls. Control samples were obtained from participants of a similar age group to FPIES participants, ranging from 13 to 25 months (obtained from de-identified samples collected for blood lead testing) (Supplememtary Fig. 2 A). We observed that FPIES participants exhibited heightened responses to LPS and to safe food treatments, relative to non-FPIES controls, by inducing significantly higher expression of the 9-gene iMCRP panels (Fig. 3A). This indicated the possible existence of an inherent myeloid cell “hyper-responsiveness defect” in FPIES participants. While baseline iMCRP responses in FPIES participants were elevated, the responses to FPIES triggers were, in turn, significantly higher than the responses to safe foods (Fig. 3B, C).

Since the activation potential by LPS was variable between participants (Fig. 2A), indicating variable activation levels between participants, we normalized the food responses to LPS and identified a threshold expression value of 29.24, below which the likelihood of a food being safe was 98.5% (Fig. 3B, C; horizontal green line), which was equivalent to an NPV value of 98.5 (Fig. 3D). As it could potentially cause harm to introduce response-triggering foods in the context of a pilot clinical trial, all the trigger foods were documented retrospectively from FPIES reactions that had occurred prior to enrollment in this study. Three trigger foods exhibited low signals, under the threshold of 29.24 (Fig. 3B, C), which could have been due to an inaccuracy in initial clinical classification (for example, upon review, these 3 foods had been confounded with other trigger food introductions at the same time as the reaction), or due to inaccuracy of the assay due to the existence of other unidentified pathways mediating food-mediated reactions. As shown in Fig. 3C, false positives were also prevalent, and hence the assay could not identify trigger foods, as it yielded low positive predictive values (PPV) (Fig. 3E). As such, response values over 29.24 were assigned as “inderminate” rather than “predicted triggers”. The goal of the analyses was therefore to solely assess the association of the lack of response ex vivo to safe food ingestion in vivo, which would serve useful for safe food prediction. The area under the curve (AUC) for the ROC curve was 73.16 (p < 0.0001) (Fig. 3F), which was negatively influenced by the low PPV. Hence, low signals in this assay were strongly associated with safe food identification, but high values were indeterminate. However, the identification of a number of safe foods with high specificity via the use of NPV was highly advantageous for the FPIES participants, as shown in the following section.

### Improved outcomes in participants following assay predictions relative to standard of care

**Fig. 4:**
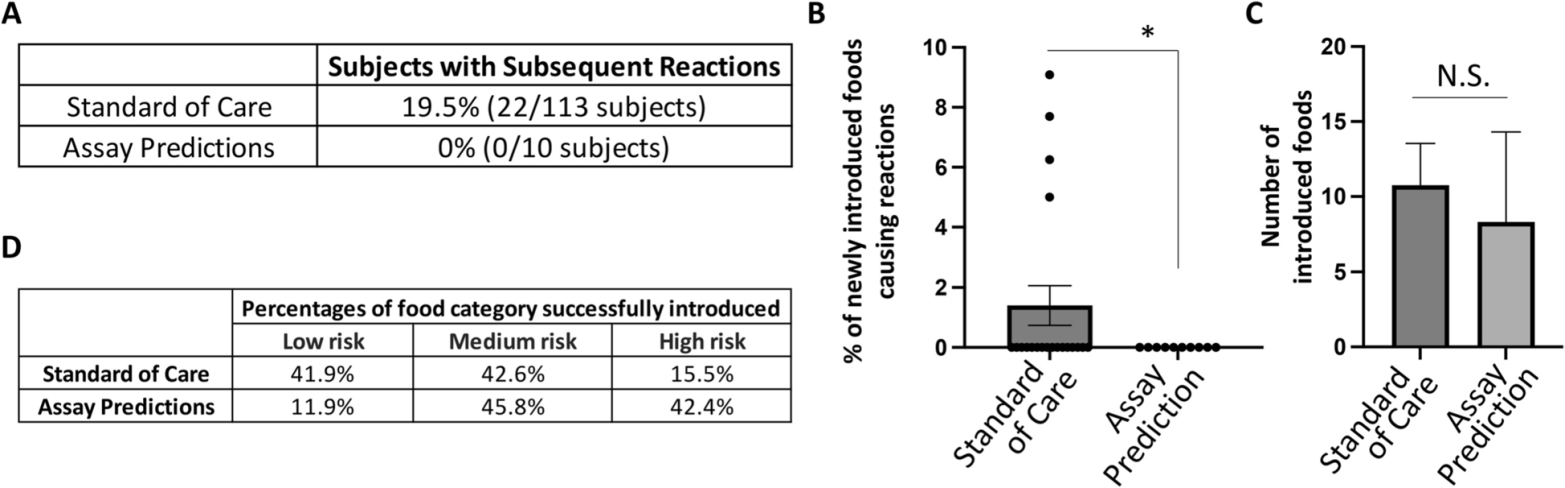
Improved outcomes in participants following assay predictions relative to standard of care. **A** Percentage of participants with subsequent reactions after first encounter with an allergist who followed the standard of care recommendations versus assay predictions. **B** Percentage of newly introduced foods causing FPIES reactions in a representative 20-participant sub-cohort (of the 113-patient cohort) who followed the standard of care recommendations relative to 10 participant cohort who followed assay predictions. * p < 0.05. **C** The number of foods introduced per patient in the 20-participant sub-cohort (following SOC) relative to 10-participant cohort (following assay predictions). **D** Percentages of successfully introduced foods by food category (low, medium, or high risk) in the representative 20 sub-cohort participants who followed the standard of care recommendations versus the 10 participant cohort who followed assay predictions

We compared the outcomes of the 10-participant cohort that followed assay predictions, relative to a 113-participant FPIES cohort that followed standard-of-care recommendations at the University of Michigan Allergy Clinic (Supplementary Fig. 2), the latter of whom were selected to match the trial group (Supplementary Fig. 2B). The demographics of the two groups were comparable in median ages for FPIES onset and first food introductions after diagnosis, numbers of triggers per patient, gender, and ethnicity (Supplementary Figs. 2B, 4). 19.5% (22/113) of the FPIES participant cohort experienced a subsequent reaction after physician and dietitian education on trigger avoidance and safe home introduction of new foods per consensus guidelines (Fig. 4A). This contrasted with a 0% reaction rate in this pilot 10-participant study following assay predictions (Fig. 4A). A more in-depth review of individual food introductions from a representative 20-participant sub-cohort of the 113 SOC cohort, revealed that the percentage of reactions to newly introduced foods was significantly lower in patients following assay predictions than SOC (Fig. 4B). The numbers of introduced foods per patient, to calculate the latter rates, were comparable (Fig. 4C). Importantly, we found that assay predictions altered the manner of food introductions to include a higher percentage of high-risk foods usually avoided under SOC recommendations (Fig. 4D), potentially leading to a more diversified diet.

### Best fit model comprising cow milk, green beans, pear and rice

We identified a best fit model comprising cow milk, green beans, pear and rice, which resulted in an ROC AUC of 93.49 (Supplementary Fig. 5 A), and an NPV and PPV of 100% at the cutoff expression value of 1743 (Supplementary Fig. 5B, C). Unlike the model that utilized our total panel of 28 foods, this model did not generate false positives, and as such requires further future validation. FPIES subjects in whom one of these 4 foods were triggers exhibited dramatically higher expression values than those in whom these foods were safe (Fig. 4D).

### Identification of spleen tyrosine kinase (SYK) to partially mediate food-stimulatory responses in myeloid cells, in a distinct manner from LPS

To identify potential pathways that mediate food-stimulatory responses that we observe in myeloid cells, and to determine whether LPS contaminants within food treatments had contributed to these repsonses, we analyzed the scRNA-Seq data from the two subjects described in Fig. 2B–G, whose peripheral WBCs were treated with safe foods, trigger foods and LPS. We sub-clustered the myeloid cells pooled from all the treatment groups from the two subjects. The reason for pooling the myeloid cells from all the treatment groups in the sub-clustering procedure was to be able to identify the similarities and differences in the responses of the treated cells between the trigger food-treated versus the LPS-treated groups, relative to baseline untreated, within the same subclustering plot. This allows for identifying, in an unsupervised manner, which cell populations were induced by trigger foods relative to those induced by LPS.

We identified 4 distinct sub-clusters (Supplementary Fig. 6 A, B) from the pooled myeloid cells from all treatment groups. We found that trigger food treatments did not induce the 9-gene panel in the same myeloid cell subclusters as LPS did (Supplementary Fig. 6B), with the induction of subcluster 2 appearing to be LPS-specific, while the induction of subcluster 4 appearing to be trigger food-specific (Supplementary Fig. 6B). This led us to screen these two subclusters for the differences in induced receptor expression between them. This screen identified one receptor, the C-type lectin Mincle (CLEC4E), which was induced in subcluster 4 in response to trigger foods, but not in subcluster 2 in response to LPS (Supplementary Fig. 6 C). Consistent with the distinctness of the induction of CLEC4E/Mincle receptor by trigger food stimulation (and not LPS) in subcluster 4, we observed that the inhibition of the C-type lectin receptor mediator, SYK [19], partially inhibited the panel expression (Supplementary Fig. 6D), whereas TLR4, TLR2 or MyD88 inhibitors, which are known to inhibit LPS-mediated induction of IL-6, failed to inhibit trigger food-induced IL-6 or the remaining genes in our panel (Supplementary Fig. 6D). Hence, this data identified that C-type lectin receptor signaling partially mediates the ex vivo reactions to food treatments, which are independent of the TLR2/4 pathways that mediate LPS signaling.

## Discussion

FPIES is understudied and primarily afflicts a vulnerable population [20]. The FPIES diagnosis carries long-term risks that are not always well-addressed by the current approach to food introductions among patients with multiple trigger foods [21]. Most extant FPIES studies are limited to surveys, observational data, or clinical FPIES challenge results [17, 18, 22–24]. While these studies are important in the understanding of FPIES, experimental ex vivo models of how food antigens interact with and stimulate immune cells are needed to advance the field. Our study provides the first evidence that iMCRPs could serve as an ex vivo FPIES model. The study provides insight into a novel strategy for potentially identifying safe foods for FPIES patients based on negative predictive value obtained from testing in the ex vivo model.

The iMCRP signals we identified are relevant to previously reported in vivo FPIES reactions. We observed considerable overlap in the genes that we identified as induced in the myeloid cell sub-cluster ex vivo and those that are reported to be induced in the peripheral blood of patients during FPIES reactions, such as IL-6, CCL20, TNF, IL-8, and IL-10 [17, 18]. Indeed, a fundamental question posed by FPIES is how the innate immune response, which is thought to be one of the major responses underlying FPIES, could vary in food-specificity between individuals [17, 18]. Members of the FPIES field have suspected that the general inability to reliably detect antigen-specific T cell responses in FPIES might be due to tissue residence of antigen-specific T cells in FPIES [18, 20]. The present results, however, suggest that perhaps a crucial part of the FPIES pathogenic response may be related to myeloid cell responses to specific foods. This is an intriguing possibility and warrants further study.

The present study has limitations. First, the occurrence of false positives may lead to the unfavorable outcome of unnecessarily leading patients to avoid certain safe foods. Hence, it is important to explicitly assign the foods that elicit high signals as “indeterminate”, and not as “predicted triggers”. The utility of the present assay approach, if further validated, would solely rely on negative predictive value in predicting safe foods to jumpstart reaction-free food introductions during initial food trialing. This could be very helpful since safe food identification is challenging in the initial stages of the disease when food repertoires are extremely limited. Early life is a key time when food aversions may develop, which could increase the likelihood of failure-to-thrive. Second, given the variety of foods we sought to test, we employed whole food homogenates for ex vivo testing. This could raise the possibility of food contamination, such as with endotoxin. However, the reproducibility of the responses (to the same food treatments) between different blood draws taken at different time points from the same individual, and the lack of TLR inhibitors’ ability to inhibit the iMCRPs to direct food antigen stimulation, both indicate that these responses may be less likely to be due to endotoxin contamination [25, 26]. Future work may require comparisons between purified food proteins and the present approach. Third, other food components, aside from endotoxin, might be responsible for the high degree of false-positives in the ex vivo model. It is important to note that the food homogenates used were not digested as they would be in the stomach in in vivo before reaching the duodenum. Therefore, future studies using pre-digested foods with gastric enzymes may address the false positive rates obtained in this assay. Moreover, future studies investigating the specific identity of the moieties that trigger FPIES may also contribute to improving trigger food prediction and PPV for this assay. Fourth, this work included a pilot clinical trial that did not deploy randomization. Given the pilot nature of the trial, this was appropriate, but further work will need to address this. Fifth, we were also unable to prospectively test trigger food predictions with oral food challenges due to the perceived risk of such an action. Future work may require such challenges, depending on the risk level and benefit expected. Sixth, this preliminary study remains under-powered to be able to analyze the association of the induction of each gene – within the 9-gene panel – to the occurrence of the FPIES reactions as well as the severity of those reactions. These analyses can be the subject of future larger follow-up studies. Seventh, this study focusses on early childhood FPIES, does not address later childhood FPIES or adult FPIES, and only focusses on acute and not chronic FPIES. These limitations can be the subject of future investigations. Eighth, aside from the data on mincle and the SYK inhibitor, the other cluster of iMCRPs was without an identified receptor, so further work may require additional investigations in this regard. Lastly, the RNA-based method requires extensive labor and reagent inputs, making any future scaling of this specific method difficult. However, there are methods, using various protein-based assays, to address this in the future.

Like other understudied disorders, FPIES warrants intense investigation not just for the sake of those afflicted, but also to reveal crucial insights into normal physiology, common mechanisms, and key pathways [27]. The authors hope that the work contained herein serves as a partial answer to the clarion calls (21, 28) to address the FPIES community’s needs through better diagnostics. This work can lay the foundation for a new approach to understanding FPIES pathophysiology, developing FPIES diagnostics, and perhaps ultimately improving care for patients with this disorder. By developing our ex vivo model of FPIES-like food responses, we can help begin to define the genetic and immune underpinnings of individual-specific food responses. Indeed, this initial evaluation of how ex vivo immune cells respond to different food antigens, and the mechanisms involved, can lay a foundation for future development of more definitive approaches to understanding FPIES pathogenesis, diagnosis, and perhaps treatment.

## Conclusions

iMCRPs represent a novel and potentially useful tool that associates with safe food ingestion in FPIES patients for foods that fail to elicit immune cell reactions.

## Supplementary Information

Supplementary material 1

Supplementary material 2

## Data Availability

The sc-RNA-Seq data presented in this study has been deposited in NCBI's Gene Expression Omnibus (GEO): https://www.ncbi.nlm.nih.gov/geo/query/acc.cgi?acc=GSE232839.

## Abbreviations

FPIES: Food protein-induced enterocolitis syndrome
iMCRPs: Individual-specific myeloid cell reactivity patterns
SOC: Standard-of-care
IRB: Institutional Review Board
LPS: Lipopolysaccharide
NPV: Negative predictive value
PPV: Positive predictive value
ROC: Receiver operating characteristic
AUC: Area under the cluster ROC curve
CI: Confidence interval
WBCs: White Blood Cells
PCA: Principal component analysis
scRNA-Seq: Single cell RNA sequencing
SYK: Spleen tyrosine kinase
