# Supplementary material for: Association of myeloid cell reactivity patterns with safe food predictions in FPIES patients
Source: Allergy Asthma Clin Immunol. 2025 May 21;21:24. doi: 10.1186/s13223-025-00968-1 (PMC12093898; doi:10.1186/s13223-025-00968-1)
Supplement: Supplementary file 1 — Supplementary material 1 [file 13223_2025_968_MOESM1_ESM.docx]

**A NOVEL BLOOD TEST TO PREDICT SAFE (NON-TRIGGER) FOODS FOR INFANTS AND TODDLERS WITH FOOD PROTEIN-INDUCED ENTEROCOLITIS SYNDROME (FPIES).**

**PRINCIPAL INVESTIGATOR:** Mohamad El-Zaatari PhD. Research Assistant Professor, Division of Gastroenterology, Department of Internal Medicine, University of Michigan.

**CO-INVESTIGATORS:**

*i)* Charles Schuler MD. Assistant Professor of Internal Medicine, Division of Allergy and Immunology.

*ii)* Georgiana Sanders MD. Professor of Internal Medicine, Associate Professor of Pediatrics and Research Associate Professor of Mary H Weiser Food Allergy Center, Medical School, University of Michigan.

*iii)* John Y. Kao MD. Associate Professor, Division of Gastroenterology, Department of Internal Medicine, University of Michigan.

**I. PROTECTION OF HUMAN SUBJECTS**

**1. *Risks to Human Subjects***

**A. Human Subjects Involvement, Characteristics, and Design**

*Describe and justify proposed human involvement*

The **objective** of this project was to validate a blood test that can identify safe foods for protein-induced enterocolitis syndrome (FPIES).

**Background:** FPIES is an allergic disease that leads to repetitive profuse vomiting in response to solid food. In severe cases, when patients fail to thrive, FPIES requires nasogastric tube installment or total parenteral nutrition (TPN). The major challenge in managing FPIES is the identification of safe (non-trigger) foods in a timely manner, while avoiding repetitive allergic reactions and recovery periods. This study proposes a solution to the problem by developing a new blood assay that screens a large number of foods (more than 20) in a culture plate. Its purpose is to rapidly predict what the safe foods are. The outcome is to overcome the time-lapse required to identify such safe foods, which could normally extend past a year or longer, by which time the child may have failed to thrive or developed food aversions due to association with negative experiences.

FPIES leads to repetitive hospital and ER visits with 2,064 encounters occurring at the University of Michigan Health System (UMHS) during the initial screening period, prior to the initiation of the study, between 11/01/17 until 11/01/18 (data obtained using Datadirect for cohort discovery results; FPIES ICD10 K52.2). The latter number of encounters only comprised newly diagnosed patients.

In this study, FPIES patients were recruited from the UMHS patient cohort. The plan was to enroll 10 subjects with FPIES exhibiting reactions to 2-3 foods or more. Recruitment took place at the University of Michigan Allergy Clinics, and University of Michigan Pediatric outpatient clinics.

**Hypothesis:**

The blood test that we developed identifies safe foods for FPIES patients.

**Specific aim**:

Assess the assay’s precision by its predictive accuracy in identifying safe foods.

**Exploratory aim:**

Gain further insight into the pathophysiology of FPIES through single cell RNASeq.

**Statistical Design:**

A random-effects logit model was used to model the binary outcome (safe or trigger food) as a function of the 9 biomarker measurements in the assay. The random effect in the logit model took into consideration of the correlated data measured within the same subject. A cluster receiver operating characteristic (ROC) curve analysis was used to assess the precision of the assay. Specifically, we computed the area under the cluster ROC curve (AUC), along with a 95% confidence interval (CI). The assay was predictive if the lower limit of the 95% CI is above 0.5, which is the null value indicating no predictive ability. A threshold expression value of 29.24, of the sum of fold changes of the 9-gene panel (normalized to LPS induction), under which the negative predictive value (NPV) was determined to be 98.5%, was computed and utilized.

**Brief description of the proposed study:**

FPIES is a severe food allergy to a wide variety of foods in babies and toddlers. Usually, the symptoms resolve between the ages of 3 to 5 years old. However, the children are at risk of failing to thrive, and requiring a nasogastric tube or TPN. The objective of the study was to validate a newly developed blood assay that was hypothesized to rapidly identify a number of safe (non-trigger) foods for the affected children.

**Subject Recruitment:**

Subjects were recruited through the University of Michigan Allergy Clinics, and University of Michigan Pediatric outpatient clinics:

1. Patients were referred by the clinician caring for the patient.
2. Pre-screening by reviewing FPIES visits in the past 2 years was performed. Data Direct, EMERSE and billing codes were used to identify potential subjects. The patient's primary allergist or pediatrician were informed about study team’s contact with the identified patients.
   - Patients were then be contacted by email and/or telephone by the clinical members of the study team to explain the study and determine whether they are interested in participating. Opt out information was provided in the email and by phone contact.
3. Patients were able to self-refer:
   - The study will be listed in UMHealthResearch.org
   - Flyers describing the study, with study team contact information, were posted in the participating clinics.

Inclusion Criteria:

1. Patients aged with a median age of 0.75 years, ranging from 0.5 to 2.25 years old, with 2 or more trigger foods with recurrent delayed vomiting, were recruited.

Exclusion Criteria:

1. Patients without a physician diagnosis of FPIES.
2. Patients who were currently on medications that suppress the immune system.
3. Patients who did not have at least 2 trigger foods identified.
4. Patients who had a history of an organic GI disease (e.g., inflammatory bowel disease, celiac disease, biliary disorders, bowel resection), cardiac, pulmonary, neurologic, renal, endocrine, or gynecological pathology
5. Lack of parental or guardian informed consent.

Study Design

1. Potential participants were contacted by email and / or telephone after pre-screening of physician-diagnosed FPIES patients who had visited the UM allergy clinics within the past two years of the study, or who were in contact with the UM allergy clinic nutritionist. An option for the patients to opt out was be provided. The patient's primary allergist or pediatrician was informed about the study team’s contact with the identified patients. Some patients were also referred by their primary allergist during a clinic visit.
2. If a parent/guardian expressed interest in the study, they were sent/given a copy of the informed consent to review.
3. An appointment was scheduled with a study team member at the UM allergy clinics.
4. The protocol was discussed face-to-face with parent/guardian with an opportunity to ask questions and review the informed consent document. After written informed consent was signed by one parent/guardian, study procedures began. Parent/guardian were given a copy of the signed informed consent and it was imaged into the chart.
5. Parent/Guardian were asked to fill out Questionnaire One at the time of entry into the study.
6. Parent/Guardian were instructed regarding scheduling the blood draw for analysis:
   1. Only known “safe foods” (foods that do not induce repetitive vomiting) were consumed for one week before blood draw.
   2. Blood draw was performed at the MLabs Blood Draw Stations at Michigan Medicine outpatient clinics.
7. Patient had 6 ml of blood drawn for analysis:
   1. 5.5 ml of blood for testing of potential food reactions
   2. 0.5 ml of blood for RNA/DNA analysis, which were stored.
8. After blood draw, diet was managed per the primary clinician and nutritionist.
   1. Parents were given Questionnaire Two to fill out detailed information about all foods introduced until the results of the assay were received.
   2. During that time, food introduction were continued as directed by primary allergist and/or nutritionist
9. Blood was analyzed by the PI in a research lab at the University of Michigan to determine differences in the PI’s assay between potentially tolerated and not tolerated foods (see description of laboratory test lines 164-175).
10. After identification of potentially “safe foods” to be introduced, the information was given to the clinical study team and child’s clinician, who contacted the parents by phone or email (parental preference).
    1. Parents were asked to detail the current nutritional status at the time, including foods tolerated or not tolerated since the initial blood draw. Results were recorded by the study team and the parents were asked to return Questionnaire Two.
    2. After discussion within the clinical team (including clinical research team and child’s clinicians), parents were instructed to introduce new foods identified as potentially safe into the diet, one at a time for seven days.
    3. Results of these trials were recorded on Questionnaire Three and returned to the study team within 3 months.
11. A second blood draw was performed, at least 2 weeks after the initial blood draw. 6 cc were drawn:
    1. To explore the consistency of a standard panel of foods vs individually prepared foods
    2. To perform additional analyses including single cell RNASeq (scRNA-seq) on WBCs following treatment with foods.

Study Grid

**B. Sources of Materials**

The research material obtained from the human subjects

1. 6ml of blood in a K2 EDTA (lavender top) blood tube were withdrawn by specialized phlebotomists in the blood draw unit. White blood cells (WBCs) were isolated from 5.5 ml of blood using ACK buffer lysis of red blood cells, within 1 hour of blood collection. Blood was kept on ice in the K2 EDTA tubes during the waiting period (< 1 hour). WBCs were plated in 24 well plates, and exposed to different food homogenates for 3 hours. Total RNA was extracted, and RT-qPCR for our gene panel was used to generate the heatmap.

A second blood draw was requested from a limited number of subjects, at least 2-4 weeks after the initial blood draw, for scRNA-seq. In this case, the blood was prepared in the same manner as for the initial draw above, but instead of extracting RNA, samples were submitted to the UofM Advanced Genomics Core for them to perform scRNA-seq on the samples. The data were analyzed using the cloupe software by 10x Genomics.

2. At the termination of the protocol samples and data ceased to be accessed, or otherwise accessed again after IRB approval transferred to another existing protocol. There were no plans to destroy records or samples.

Protection of Information:

All data was kept on REDCap, a password-protected, HIPAA compliant, web-based application developed by Vanderbilt University to capture data for clinical research and create databases and projects. The databases use instruments such as surveys and forms as research capture tools. Projects are self-sufficient and secure databases that can be used for normal data entry or for surveys across multiple distinct time points. Only study team members had access to this data.

1. A master list that includes identifiable data was kept in a REDCap “Study Housekeeping” database that included subject number, subject name, parent names, MRN, contact information including address, email, phone number, documentation of blood draw, samples saved and filling out of questionnaires.
2. All data variables will be kept in a separate “Research Data” REDCap database. Subjects were identified only by subject number in this database. Data included:
   1. Results of blood tests
   2. Questionnaire answers
   3. Age
   4. Number of food reactions
   5. Identified trigger foods
   6. Identified tolerated foods.
3. Data downloaded for analysis had only subject number included.
4. Stored RNA, DNA or cDNA samples contained only the subject number without personal identifiers.
5. Parents/guardians were given the option to request that stored blood samples be destroyed at the end of the study.

**C. Potential Risks**

Describe potential risks

The risk in this study could have arisen due to incorrect predictions by the test. The current test being validated was not an FDA-approved or cleared test. However, current standard of care recommended which foods to trial in a random fashion, and incorrect predictions frequently occurred leading to reactions. Hence this study did not pose an added risk to the current standard of care. The indication of what to trial was communicated to the patients by the allergist and nutritionist in an analogous manner to the standard of care. However, the only difference is that the allergist and nutritionist in this study used this assay for indication regarding what foods to trial. Even though it did not occur in this study, but if a food were falsely identified as safe by the assay but triggered a reaction, then these reactions would have been managed by the nutritionist and allergist as per standard of care for failed trials.

Blood draw from infants and toddlers was a safe procedure with minimal risks. Trained and experienced phlebotomists at UMHS perform blood draws on infants and toddlers routinely. The procedure may have cause anxiety for the infants / toddlers. The test could have resulted in a small bruise or mild soreness for the child at the site of blood draw. The bruise could have lasted for a few days.

A risk of breach of confidentiality always exists in all studies.

Describe alternate treatments and procedures

The parent / child could have proceeded with introduction of new foods per guidance of the nutritionist. This is based on historically better tolerated foods in the general FPIES population and may or may not have applied to the patient.

**2. Adequacy of protection against risks**

**A. Recruitment and Informed Consent**

Plan for recruitment

All FPIES patients were recruited from the University of Michigan Allergy Clinics, and University of Michigan Pediatric outpatient clinics. The patients were identified by pre-screening of the UM FPIES patient cohort. Pre-screening was performed by members of the Division of Allergy and Immunology at Michigan Medicine. Patients were not contacted by the study team until pre-approval was obtained from the primary allergist or pediatric clinician. Suitable FPIES patients who fulfilled the inclusion and exclusion criteria were contacted by email and / or phone. An option to opt out was provided. If subjects were interested in participating, a meeting with the study team was scheduled at the UM allergy clinics. The patients were presented with the consent form by the study team member to consider consenting for the study and the blood draw procedure. Patients were told that their decision whether to participate in the study would not affect their clinical care.

Describe the circumstances of consent

A study coordinator or clinical study team member familiar with the study protocol obtained informed consent for all participants. Consent/assent was obtained prior to any study measures/questionnaires. Each participant received a verbal and written explanation of the purposes, procedures, risks, and potential benefits of the study in language appropriate for the individual.

**B. Protections Against Risk**

Planned procedures for protecting against risk

1. The risk of a false result were to be managed by the allergist and nutritionist as per standard of care in managing reactions to failed trials.

2. The risk from blood draw were minimal as they are routinely performed by the phlebotomists at UMHS.

3. All data variables were kept in a separate “Research Data” REDCap database. Subjects were identified only by subject number in this database.

4. A separate “Housekeeping” REDCap database was used to link the patient identifiable information and the study number.

**3. Potential benefit of the proposed research to the research subjects and others**

Potential benefits

There may have been direct benefit to the patient by the potential of identifying tolerated foods, thus avoiding the adverse reactions of frequent emesis and potential dehydration, as well as reducing the trauma parents encountered when giving their infant a food that causes such reactions. There may have been benefit to the larger community of FPIES patients if this protocol indicated that the new assay could identify tolerated vs non-tolerated foods.

Discuss why risks to subjects are reasonable in relation to benefits

Patients already encountered incorrect predictions and failed trials with allergic reactions regularly. The indication from the assay was expected to predict safe foods at a higher rate than the guesswork utilized in the current standard of care.

**4. Importance of the knowledge to be gained**

Discuss importance of the knowledge to be gained

Validation of this test had the potential to modify patient care strategy for patients with FPIES. As such, random guessing of safe foods was replaced by a scientific “guide” to what might have been hypoallergenic to the body’s immune cells using this test.

Discuss why the risks are reasonable in relation to the importance of the knowledge to be gained

Currently, trialing food is random. Obtaining a guide for what might be a safe food, by utilizing our proposed assay, would be invaluable. Incorrect predictions cannot pose more risk than random selection of foods to trial. Hence, there is only benefit to be gained if the test is predictive or partially predictive.

**II. INCLUSION OF WOMEN AND MINORITIES**

*Inclusion of Women*

This study will include children of both genders.

*Inclusion of Minorities*

The disease is overwhelmingly predominant in White Caucasian children (Based on personal communication with allergists caring for FPIES patients at UofM clinics, and the following published abstract: R. Tarrant and A. and Byrne. Clinical Presentation and Food Allergens Associated with Food Protein-Induced Enterocolitis Syndrome – a frequently misdiagnosed rare form of gastrointestinal food hypersensitivity. *European Society of Pediatric Gastroenterology, Hepatology and Nutrition* 62(1); DOI: 10.13140/RG.2.1.1621.2087). Therefore, for the purpose of feasibility of meeting the required proposed patient numbers for statistical analyses, we chose a majority of White Caucasian subjects for this initial study. If patients from other ethnicities – who did not usually have FPIES – enrolled, they would not have been excluded from the study.

*Inclusion of Children*

Children with FPIES were included in this study.

**III. PLANNED ENROLLMENT TABLE**

|  |  | | | | | |
| --- | --- | --- | --- | --- | --- | --- |
| Total Planned Enrollment: | | 20 | | | | |
|  | | | | | | |
| TARGETED/PLANNED ENROLLMENT: Number of Subjects | | | | | | |
| Ethnic Category | | | Females | | Males | Total |
| Hispanic or Latino | | | 0 | | 0 | 0 |
| Not Hispanic or Latino | | | 6 | | 4 | 10 |
| Ethnic Category: Total of All Subjects * | | | 6 | | 4 | 10 |
| Racial Categories | | |  | | | |
| American Indian/Alaska Native | | | 0 | 0 | | 0 |
| Asian | | | 0 | 0 | | 0 |
| Native Hawaiian or Other Pacific Islander | | | 0 | 0 | | 0 |
| Black or African American | | | 1 | 0 | | 1 |
| White | | | 5 | 4 | | 9 |
| Racial Categories: Total of All Subjects * | | | 6 | 4 | | 10 |
