# Supplementary material for: Association of myeloid cell reactivity patterns with safe food predictions in FPIES patients
Source: Allergy Asthma Clin Immunol. 2025 May 21;21:24. doi: 10.1186/s13223-025-00968-1 (PMC12093898; doi:10.1186/s13223-025-00968-1)
Supplement: Supplementary file 2 — Supplementary material 2 [file 13223_2025_968_MOESM2_ESM.pdf]

# SUPPLEMENTARY FIGURES

## Supplementary Figure 1

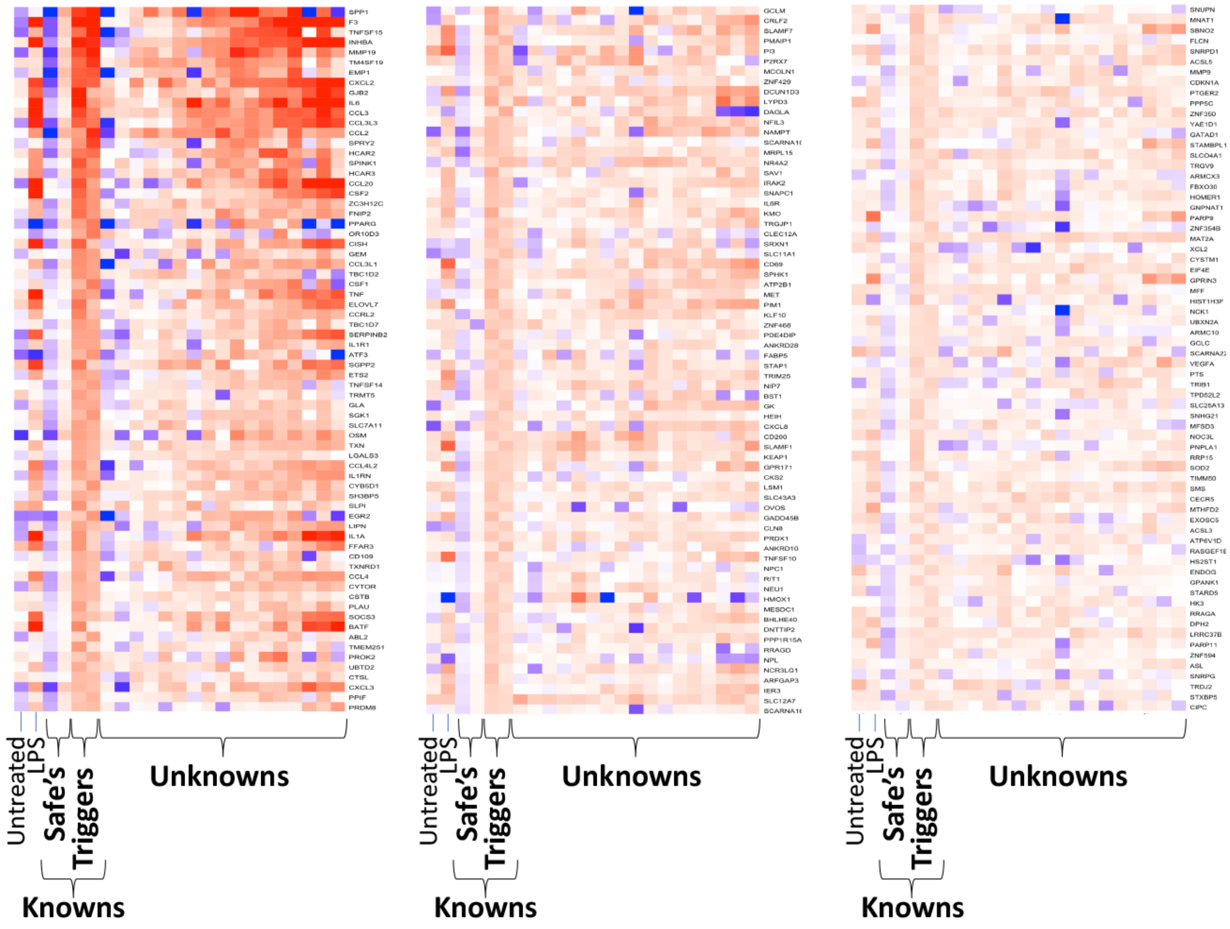


**Supplementary Figure 1.** Annotation of individual genes induced by direct stimulation of WBCs with FPIES trigger food treatments relative to safe food treatments from the FPIES participant's case study. The figure shows the microarray heatmaps of 208 genes that are induced by treating the case study patient's WBCs *ex vivo* with 2 known triggers versus 2 known safe foods. Each column represents one treatment including untreated, LPS, 2 known safe foods, 2 known trigger foods, and individual food treatments of unknown safe or trigger status.

**Supplementary Figure 2**

**A**

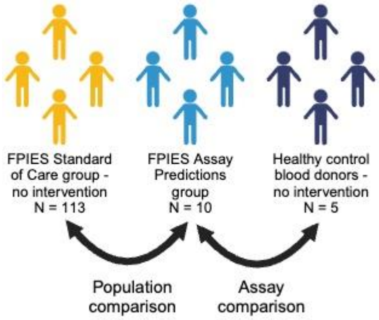


**B**

| | Age of FPIES onset, median (range) | Age of study enrollment, median (range) | Sex, N (%) | | Ethnicity | | | | |
|---|---|---|---|---|---|---|---|---|---|
| | | | Male | Female | White | Black | Asian | Other | Unknown |
| Standard of care comparison group | 6 mo (4 mo - 10 mo) | Not applicable | 51 (45) | 62 (55) | 99 (88) | 4 (3.5) | 5 (4.4) | 3 (2.7) | 2 (1.8) |
| FPIES study cohort | 5 mo (4 mo - 10 mo) | 9 mo (6 mo - 27 mo) | 4 (40) | 6 (60) | 9 (90) | 1 (10) | 0 (0) | 0 (0) | 0 (0) |

**Supplementary Figure 2. Study design and** demographics of FPIES participant cohort by age, sex and ethnicity. *(A)* Study design outlining the "FPIES Assay Predictions" group, the "Healthy control" blood donors, and the "FPIES Standard of Care" groups. (*B*) Demographics of the 113 FPIES participant cohort under the standard of care versus the 10 FPIES study cohort following assay predictions.

**Supplementary Figure 3**

| | First Blood Draw | | | | Second Blood Draw | | | |
|---|---|---|---|---|---|---|---|---|
| | Untreated | LPS | Breast Milk | Sweet Potato | Untreated | LPS | Breast Milk | Sweet Potato |
| TNFSF15 | 1 | 7.29 | 1.18 | 54.9 | 1 | 6.94 | 2.4 | 45.3 |
| SPP1 | 1 | 2.19 | 0.76 | 44.2 | 1 | 5.52 | 1.29 | 72 |
| SPINK1 | 1 | 10.7 | 1.39 | 17 | 1 | 8.88 | 0.91 | 9.42 |
| MMP19 | 1 | 383 | 0.84 | 3.72 | 1 | 633 | 2.81 | 5.21 |
| IL6 | 1 | 29.4 | 0.87 | 12.1 | 1 | 40.2 | 1.49 | 17.6 |
| CCL3L3 | 1 | 3.92 | 1.01 | 13.9 | 1 | 2.69 | 1.06 | 11 |
| CCL20 | 1 | 122 | 2.69 | 9.78 | 1 | 169 | 6.3 | 12 |
| CCL3 | 1 | 20.6 | 0.73 | 6.48 | 1 | 37.4 | 1.62 | 12.4 |
| CXCL2 | 1 | 30.3 | 1.13 | 10.4 | 1 | 24.8 | 2.23 | 6.39 |

**Supplementary Figure 3.** Consistency of the food-induced WBC responses between two different blood draws from the same subject. Heatmap of the 9-gene RT-qPCR panel in response to breast milk (safe food) versus sweet potato (trigger food), relative to untreated versus LPS treatments, from two blood draws withdrawn on two different dates, one month apart, from the same subject.

**Supplementary Figure 4**

| | Standard of Care | Assay Predictions |
|---|---|---|
| Number of triggers, median (range) | 2 (2-7) | 2 (2-4) |
| Number of triggers, N (%) | | |
| 2 | 57 (50) | 7 (70) |
| 3 | 29 (26) | 2 (20) |
| 4 | 20 (18) | 1 (10) |
| 5 | 5 (4.4) | 0 (0) |
| 6 + | 2 (1.8) | 0 (0) |

**Supplementary Figure 4.** Consistency in the number of triggers per patient between the 113 FPIES participant cohort under the standard of care and the 10 FPIES participant cohort following assay predictions.

**Supplementary Figure 5**

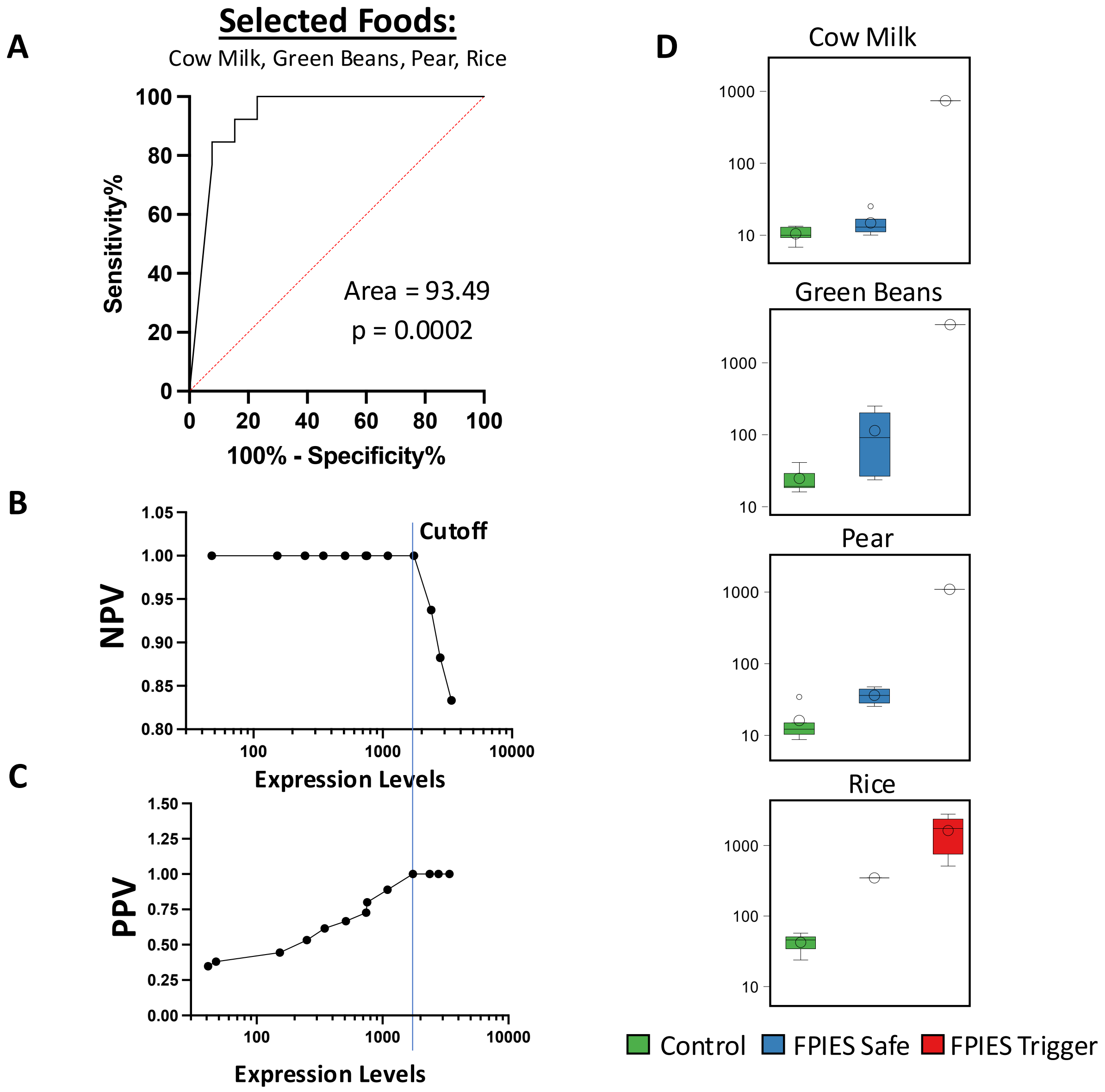


**Supplementary Figure 5.** Computation of a 4-food best fit model with high negative and positive prediction values. (*A*) ROC, (*B*) NPV, and (*C*) PPV of a 4-food best fit model computed based on generalized linear mixed models with random intercept for participant (*e.g.*, binary logistic regression with random intercept). Significance was based on $p < 0.05$. Inclusion of these foods was based on undjusted analyses and box plots. (*D*) Relative expression values of the 9-gene panel

in each of the 4 foods from the best-fit model in participants for whom the food was safe versus those for whom the same food was a trigger, relative to non-FPIES controls.

## Supplementary Figure 6

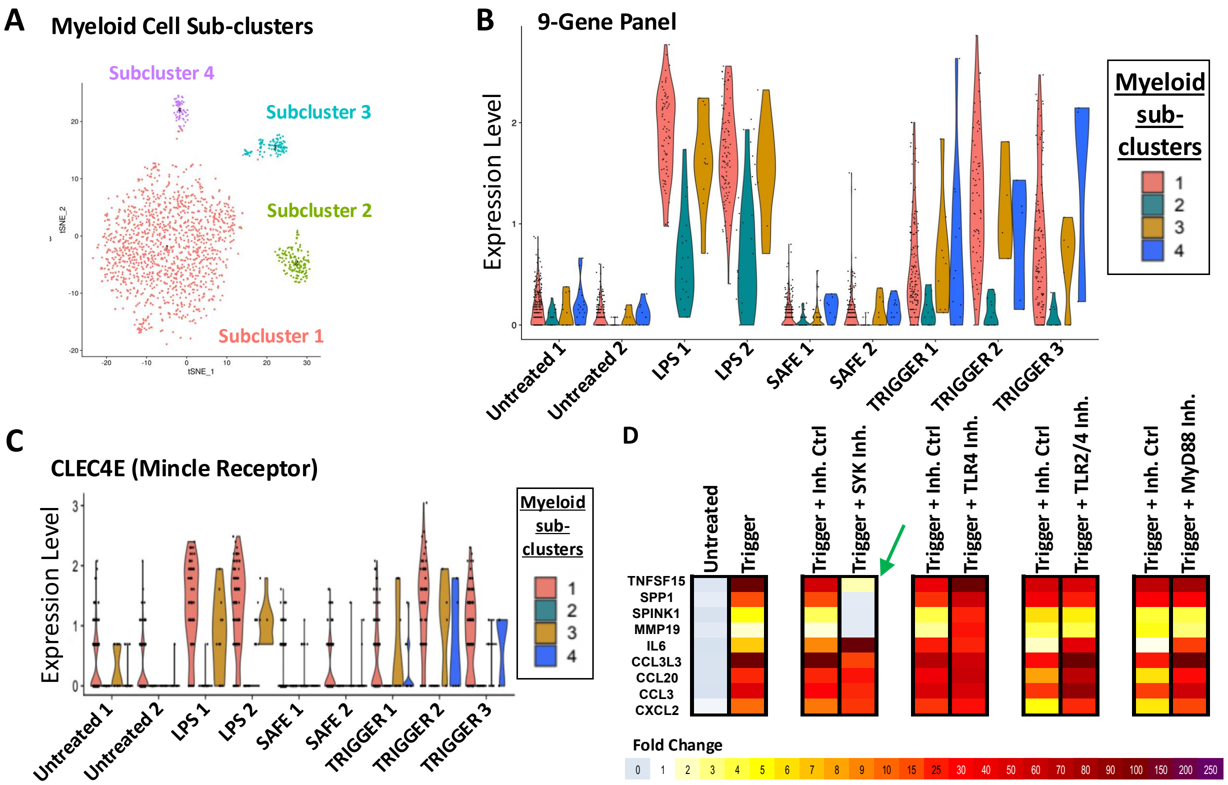


**Supplementary Figure 6.** Myeloid cell responses to food treatments, partially mediated by SYK, are distinct from LPS-induced responses. (*A*) Sub-clustering of the myeloid cell cluster from WBCs into 4 subclusters based on variable genes (n=3000) calculated from the transcriptome. (*B*) Violin plots of the expression of the 9-gene panel in peripheral blood WBCs in response to safe vs trigger foods (compiled from two different participants) relative to untreated and LPS-treated WBCs from the same participants. (*C*) Violin plots of CLEC4E (Mincle receptor gene) expression in peripheral blood WBCs in response to safe and trigger foods relative to untreated and LPS-treated WBCs. (*D*) Heatmap of the 9-gene RT-qPCR panel in response to trigger food treatment with or without pre-incubation with the inhibitors of SYK, TLR4, TLR2/4, or MyD88, relative to inhibitor controls and untreated. The green arrow annotates the inhibition of the upper cluster (comprising TNFSF15, SPP1, SPINK1, and MMP19) by SYK inhibitor.
